# Is there a need of monkeypox vaccine amidst the hesitancy of COVID-19 immunization in Pakistan?

**DOI:** 10.1016/j.amsu.2022.104391

**Published:** 2022-08-23

**Authors:** Zobia Ansari, Hurriyah Ramzan, Ramsha Shakeel

**Affiliations:** Department of Internal Medicine, Dow University of Health Sciences, Baba-e-Urdu Road, Saddar, Karachi, Pakistan

**Keywords:** Monkeypox virus, Vaccine hesitancy, Covid-19 vaccine, Smallpox vaccine

## 1 Main text

While the world is still in the tormenting effect of the COVID-19 pandemic, on July 23rd, 2022, WHO declared the Human Monkeypox virus (MPXV) a public health emergency of international concern [1]. Monkeypox is a double-stranded DNA virus belonging to the orthopoxvirus genus, which includes variola (smallpox), cowpox, and vaccinia viruses [2]. In 1958, the name was given after the virus's discovery in monkeys in a Danish laboratory [3]. and was first discovered in 1970 in the Democratic Republic of the Congo (DRC) in a 9-month-old infant boy. It has since become endemic across the rest of Africa [4]. The causes of transmission are particularly prevalent among males who identify as homosexual, bisexual, or have had intercourse with other men [5]. Additionally, the virus can spread through familiar bedding and clothing, as well as direct contact with sores, body fluids, and scabs [6]. The signs and symptoms of MPXV are fever, headache, back pain, myalgia, lymphadenopathy, and a maculopapular rash that grows into vesicles and whose onset varies from 5 to 21 days [7]. Monkeypox can lead to all possible complications such as bacterial infections, encephalitis, pneumonitis, and keratitis [8]. However, mortality ranging from 1% to 10% has been reported [9].

From 1st January to August 8th, 2022, the Centers for Disease Control and Prevention (CDC) received reports of 30,189 laboratory-confirmed cases of monkeypox and 5 deaths from 75 countries across all six regions. United States of America (8,933) had the most cases overall, followed by the Spain, Germany, and the UK [10]. Asia reported its first death in India. India has recorded total 9 cases till 8th Aug, 2022 [11]. Fortunately, in Pakistan, according to National Health Services (NHS), no case of monkeypox has been reported to date, but due to the rise of the monkeypox virus in non-endemic countries, national and provincial health authorities were prompted to issue a high-level alert because the virus could also spread in Pakistan [12]. According to the CDC, there are no specific therapies for people with the monkeypox virus, and supportive care is usually sufficient. Minor outbreaks, on the other hand, have been managed with smallpox vaccinations, antivirals (tecovirimat, cidofovir, or brincidofovir), and vaccinia immune globulin (VIG). Prior vaccination with the smallpox vaccine appears to have an 85% protective effect against the monkeypox virus and may alleviate clinical symptoms of illness [13]. The JYNNEOSTM vaccine was approved by the US Food and Drug Administration (FDA) in September 2019 for the prevention of smallpox and monkeypox illnesses in individuals 18 years of age or older who have been assessed to be at high risk of infection [14]. On June 15, WHO monkeypox guidance declared that the supply of vaccines to prevent monkeypox was very constrained and also encouraged countries that did already have them to share them with other countries with no or low supply [15].

Vaccine hesitancy is one of the WHO's top ten global health challenges [16] and if we emphasize the Polio vaccine or even now COVID-19 immunization, vaccine hesitancy is still a problem in Pakistan. [Shaukat & Jafar, 2020]. Although Pakistan hasn't yet recorded a definite case of monkeypox, the virus's spread is practically certain, and yet imposing the MPVX immunization will be difficult amidst the COVID-19 vaccine hesitancy [17,18]. Due to a dearth of finances, Pakistan's healthcare sector had several challenges during the COVID-19 pandemic. It was exceedingly challenging for hospitals and healthcare organizations, to guarantee the availability of sufficient staff, ventilators, medical personnel, hospital beds, and laboratory equipment [19]. Monkeypox is endemic in many countries, thus screening for the virus through PCR at airports should be required, and cases that are suspected or confirmed must be isolated for a prodromal period. The samples are taken from nasopharyngeal swabs, ulcers, or dry swabs of unroofed lesions, and serum and tissue samples [20]. Pakistan's Healthcare agencies must ensure that the general public is made aware that the vaccine has gone through all required development steps, with no compromise on quality. Healthcare professionals have a significant role in counteracting doubts because their suggestions help in the acceptance of the vaccine. Educational campaigns targeting medical professionals should be encouraged to reduce the threat of vaccine hesitancy. Also, given the load imposed by the COVID-19 epidemic on Pakistan's already strained healthcare system, the appropriate health authorities must take a proactive stance and launch widespread awareness programs that highlight the significance of good hygiene habits, self-quarantining, and other pertinent safety precautions to prevent a monkeypox-like epidemic in Pakistan. Pakistan Healthcare agencies must ensure that the general public is made aware that the vaccine has gone through all required development steps, with no compromise on quality. Healthcare professionals have a significant role in counteracting doubts because their suggestions help in the acceptance of the vaccine. That's why It is beneficial to support educational initiatives to lower the risk of vaccine hesitancy.

Therefore, more well-organized public health programs and educational strategies are needed by the government of Pakistan to disseminate accurate information about the treatment and immunization against the monkeypox virus, and this might significantly affect the resolution of vaccine hesitancy problems. Given that many patients infected with the monkeypox virus have a moderate, self-limiting disease course, the prognosis for monkeypox may vary based on factors such as prior baseline health state, immunization status, and co-morbidities. Consequently, it seems acceptable to personalize therapies depending on each patient's probability of contracting a serious illness.

## Ethical approval

This article did not involve patients; therefore, no ethical approval was required.

## Funding sources

There was no source of funding for this article.

## Author statement

Zobia Ansari: conception of the study, drafting of the work, final approval and agreeing to the accuracy of the work. Hurriyah Ramzan: conception of the study, drafting of the work, final approval and agreeing to the accuracy of the work. Ramsha Shakeel: conception of the study, drafting of the work, final approval and agreeing to the accuracy of the work.

## Registration of research studies

1. Name of the registry: Not applicable
2. Unique Identifying number or registration ID: Not applicable
3. Hyperlink to your specific registration (must be publicly accessible and will be checked): Not applicable.

## Guarantor

The Guarantor is the one or more people who accept full responsibility for the work and/or the conduct of the study, had access to the data, and controlled the decision to publish.

Zobia Ansari, Ramsha Shakeel.

## Consent

This study was not done on patients or volunteers, therefore no written consent was required.

## Declaration of competing interest

None.
